# Factors Influencing Oral Health Behaviours, Access and Provision of Dental Care for Autistic Children and Adolescents in Countries with a Very High Human Development Index: Protocol for a Mixed Methods Systematic Review

**DOI:** 10.3390/ijerph182312346

**Published:** 2021-11-24

**Authors:** Jo Erwin, Martha Paisi, Robert Witton, Sarah Neill, Lorna Burns, Isaac Vassallo, Abigail Nelder, Jemma Facenfield, Urshla Devalia, Tara Vassallo

**Affiliations:** 1 Peninsula Dental School, University of Plymouth, Drake Circus, Plymouth PL4 8AA, UK; martha.paisi@plymouth.ac.uk (M.P.); robert.witton@plymouth.ac.uk (R.W.); lorna.burns@plymouth.ac.uk (L.B.); 2 School of Nursing and Midwifery, University of Plymouth, Drake Circus, Plymouth PL4 8AA, UK; sarah.neill@plymouth.ac.uk; 3 Peninsula Dental Social Enterprise, Plymouth Science Park, Research Way, Plymouth PL6 8BT, UK; abigail.nelder@plymouth.ac.uk (A.N.); jemma.facenfield@plymouth.ac.uk (J.F.); 4 School of Engineering, Computing and Mathematics, University of Plymouth, Drake Circus, Plymouth PL4 8AA, UK; isaac.vassallo@students.plymouth.ac.uk; 5 Royal National ENT and Eastman Dental Hospital, London University, 47-49 Huntley St, London WC1E 6DG, UK; urshla.devalia@nhs.net; 6 Plymouth Institute of Education, University of Plymouth, Drake Circus, Plymouth PL4 8AA, UK; tara.vassallo@plymouth.ac.uk; 7 National Autistic Society—Plymouth & District Branch, Plymouth PL1 4QU, UK

**Keywords:** systematic review, autism, dental care, oral health, access to health care, health care delivery

## Abstract

Autistic children and adolescents are at high risk of dental disease and experience oral health inequalities. They consistently show high levels of unmet needs in relation to their oral health and access to dental care. There are no systematic reviews that bring together the evidence on the factors that influence oral hygiene behaviours, and access to and provision of dental care for autistic children and adolescents. A systematic search will be carried out in eight international databases and in grey literature of qualitative, quantitative and mixed method research studies from countries with a High Development Index which relate to oral health behaviours, and access to and provision of dental care. Only studies where participants are autistic children and adolescents aged 19 years or under, parents/guardians/caregivers, support staff, or oral health care providers will be included. Quantitative and qualitative data will be synthesized together through data transformation using a convergent integrated approach. Thematic synthesis will be used to carry out an inductive analysis of the data. The findings from the systematic review which this protocol generates will be used in the development of an appropriate local clinic care pathway for autistic children/adolescents and to inform national policies and practices. Prospero registration: CRD 42021248764.

## 1. Introduction

Autism is a neurodevelopmental condition which affects individuals in very different ways and to different extents. Autism, a spectrum, is diagnostically characterised by persistent difficulties with social interaction and communication, and restricted, repetitive patterns of behaviours [1]. Autism is not a learning or intellectual disability; however, autistic children have a higher prevalence of intellectual disability than the general population [2], and approximately 20–30% of people with a learning disability are also autistic [3] (here we refer to the definitions of learning and intellectual disability used by the UK Department of Health and NHS) [4]. Autistic people may experience altered sensory responsivity across all eight senses. This includes under or over sensitivity resulting in sensory seeking or avoidance behaviours in different contexts. They are also likely to experience higher levels of general anxiety as they navigate a sensory and social world where their needs may be unacknowledged and unmet. It is estimated that worldwide, approximately one in every 160 children is autistic, although this is acknowledged by the World Health Organisation to be an underestimate given the lack of data on prevalence from countries in the global south [5]. A recent study of over 7 million English school pupils reported a standardised autism prevalence of 1.76% [6]. 

Autistic people are more likely than their neurotypical peers to experience comorbidity [7,8] and to have a plethora of unmet healthcare needs [9,10]. The Westminster Commission on Autism inquiry into access to health care for autistic people highlights the inequalities in access to health care for autistic people and the impact this has on their physical and mental health [11]. Some of the difficulties autistic people experience in accessing appropriate health care and support may be associated with issues such as sensory sensitivities, communication difficulties, anxiety, and social isolation [12]. Others may reflect a lack of knowledge, understanding, and adjustments by health care professionals [12]. A study from Sweden has shown that the average life expectancy of autistic people was 12 years shorter than matched controls from the general population, a deficit which increased to an average of 30 years for autistic people with learning disabilities [13], a stark indication of health inequalities. 

Most oral health problems, such as dental caries and periodontal disease, are largely preventable. However, we see in the UK and across the world that vulnerable and socially disadvantaged groups are most affected [14]. Oral health inequalities are common and reflect a range of interacting factors at the individual and societal level [14]. A systematic review found a high prevalence of dental caries and periodontal disease among autistic children and young adults, as well as poor oral hygiene levels, a significant risk factor for the development of dental disease [15]. Although the results of the individual studies on levels of dental caries are mixed, they consistently show high levels of unmet treatment needs and high levels of gingivitis [16,17]. Autistic children are also more likely than neurotypical children to receive treatment under general anaesthesia and to experience other dental problems such as bruxism, erosion, and tongue thrusting [18,19].

The high risk of dental disease among autistic children may be due to a number of factors. These include the use of tricyclic medications (a common side-effect of which is dry mouth), high and frequent consumption of cariogenic diets, poor oral hygiene, and difficulties in accessing preventative dental care [20]. Sensory sensitivities and issues around manual dexterity may also impact on the ability of autistic children and young people to brush their teeth and perform other oral hygiene routines [21]. Some may be dependent on others to provide oral hygiene for them as they may not have the skills, knowledge, or tools to be able to do this effectively. There is also evidence that autistic people encounter difficulties in accessing dental care [12]. Studies have demonstrated that attending dental visits can be a stressful experience for autistic children and their families [22]. Difficulties relating to social interaction and communication and resistance to change can make dental visits unpleasant for autistic children and create challenges for the dental team when providing care to these patients. Lack of education, training, and confidence about autism among oral health care professionals, along with the behavioural distress that these patients may experience, contribute to the reluctance of some dental health professionals to treat autistic patients [20,23]. In countries such as the UK, where public dentists have contractual budgetary restraints, the cost of adapting care to anxious patients or those needing a longer time for acclimatisation may act as a barrier [23,24].

Lack of routine dental care and preventative dental treatment is undoubtedly a significant factor for the unmet dental care needs of autistic children and young people. At present, there are no systematic reviews collating the evidence on the factors that influence oral hygiene behaviours and/or access to dental care for autistic children and young people. The aim of our research is to provide insights and evidence into improving oral health behaviours, dental experience, and access to mainstream dental services for autistic children and adolescents. The proposed systematic review will answer the following three research questions:

1. What are the factors influencing oral health behaviours in autistic children and adolescents?
2. What are the factors influencing access to dental care by autistic children and adolescents?
3. What are the factors influencing the provision of dental care to autistic children and adolescents?

### Terms and Definitions

There are a number of terms used to describe autism and there are different opinions about, and positions on, autism language [25]. People have personal preferences and individual rights to decide how they are described. Professionals, when diagnosing autism, refer to the Diagnostic and Statistical Manual of Mental Disorders (DSM-5). DSM-5 uses the diagnostic term ‘autism spectrum disorder’ (ASD) [1]. This combines all forms of the condition, including pervasive developmental disorder not otherwise specified (PDD-NOS), and Asperger syndrome. The latter was previously categorised as a separate condition and some people historically diagnosed with Asperger’s do still strongly identify as such. In this review we will adopt identity first language (e.g., autistic children) which is widely used by the autism community and is the language preference of choice of the National Autistic Society UK.

In this review we are using the term “oral health behaviours” to refer to behaviours to maintain oral health and prevent disease as identified by Public Health England [26]. These include:

1. Tooth brushing at night and on one other occasion and use of fluoridated toothpaste (1000–1500 ppm fluoride according to age).
2. Spitting out after brushing and not rinsing.
3. Brushing or supervision of brushing by parent/carer and reducing the frequency and amount of sugary food and drinks.
4. Use of sugar-free medication.

This definition will not be used as an exclusion criterion. Therefore, studies which include, for example, higher concentration fluoride toothpaste will not be excluded from the review.

## 2. Method

### 2.1. Study Eligibility

#### 2.1.1. Study Design

Qualitative, quantitative, and mixed method research studies will be included. Quantitative studies will include experimental and observational studies (randomised controlled trials, non-randomised studies, and quantitative descriptive studies). Studies of children and young people with intellectual or learning disabilities will be included if the results which pertain to autistic people are clearly distinguishable. Narrative reviews, letters, commentaries and editorials, and conference proceedings will be excluded. The Human Development Index (HDI) [27] is a summary measure of average achievement in key dimensions of human development: a long and healthy life, being knowledgeable, and have a decent standard of living (where 1.00 is the highest possible value and 0.35 the lowest). A country scores a higher HDI when the lifespan is higher, the education level is higher, and the gross national income GNI (PPP) per capita is higher. Research from countries with a very high Human Development Index [27] only will be included in the review (for a list of countries see Appendix A), the rationale being that this facilitates comparability and will support the transferability of findings to countries with advanced healthcare systems. Studies published in any language will be included. At the study eligibility and data extraction stages of the review, studies in languages other than English will be translated by the authors or members of their networks who have the necessary linguistic skills. For the benefit of completeness, there will be no restriction on the date of publication. We are aware of the speed of change in the landscape of autism, especially in relation to the change in attitudes to, and awareness of, autism in the western culture, and will be sure to contextualise the review findings in relation to the date of publication of the included studies. We will update the search to ensure that any recent studies are included.

#### 2.1.2. Inclusion and Exclusion Criteria

For a summary of inclusion and exclusion criteria see Table 1.

### 2.2. Search Strategy

#### Search Terms

The searches were developed by an experienced information specialist (LB). Literature search strategies were developed using medical subject headings (MeSH) and text words related to autism, dental care, and oral health. The electronic databases to be searched are: Embase, Web of Science, Dentistry & Oral Sciences Source (DOSS), MEDLINE, Psychinfo, Scopus, CINAHL, and SocINDEX. Grey literature will be included. Here, grey literature is defined as literature not controlled by commercial publishing organisations [28]. This will be identified by an internet search using the search engine Google, established sources of grey literature, and websites of relevant organisations including EThOS, Social Care On-line, Public Health England, Health Foundation, British Society for Disability & Oral Health, British Society for Paediatric Dentistry, National Autistic Society, British Dental Association, Autistica, National Institute for Health and Care Excellence, and Mencap. The focus on UK based grey literature reflects the fact that this review is part of a wider study which aims to inform national policies and practices in relation to the oral health care of autistic children and adolescents.

The search strategy used for the various databases and the grey literature can be found in Appendix B.

### 2.3. Data Management

A search log will be maintained to record the databases, keywords used, and results of each search.

### 2.4. Study Selection

The literature search results will be screened for duplications using EndNote and any duplications will be removed. Literature search results will be uploaded to Rayyan [29], a systematic review web application that facilitates collaboration among reviewers during the study selection process. Two reviewers (JE and MP) will independently screen all titles and abstracts against the pre-determined inclusion and exclusion criteria. Following the screening of titles and abstracts, the full texts of potentially relevant articles will be retrieved and independently screened by two reviewers (JE and MP). Should the two reviewers disagree as to whether an article/report should be included, consensus will be arrived at by discussion and/or seeking the views of a third reviewer (RW). We will seek additional information from study authors where necessary to resolve questions about eligibility. We will check the references of included studies to identify any studies that may have been missed and will consult with two experts to ensure that no relevant studies were missed.

### 2.5. Data Extraction

An extraction form will be developed and piloted before use. Data on the included studies will be extracted by two reviewers (JE and MP). Reviewers will independently extract data, and any discrepancies and disagreements will be resolved through discussion with a third reviewer (RW). For studies with missing/unclear reporting, an attempt will be made to contact the authors for further clarification. At this stage of the review, multiple reports of a single study may come to light, these will be reviewed to establish whether these multiple reports contain new data or interpretations. Where reports of the same study simply duplicate the data and interpretations from other reports of the study, they will be removed. These cases will be discussed by the reviewers as necessary.

The reasons for exclusion of full-text studies will be recorded. The PRISMA flow chart [30] will be used to present the number of studies at each stage of the systematic review. PRISMA depicts the flow of information through the different phases of a systematic review (e.g., identification, screening, and inclusion).

### 2.6. Data Items

For quantitative, qualitative, and mixed methods studies, extracted data will include study details, country, study methodology, data collection methods, participant characteristics, dental care setting, oral health setting, and outcome of interest. For qualitative studies and the qualitative components of mixed method studies, the extracted data will also include themes related to the outcomes of interest. The qualitative data, such as quotes or themes, will be extracted from the results sections of the included studies.

### 2.7. Assessment of Methodological Quality

The studies will be critically appraised by two independent reviewers (JE and MP) using the Mixed Methods Appraisal Tool (MMAT) [31]. MMAT is a checklist for appraising and/or describing studies included in systematic mixed studies reviews (reviews including original qualitative, quantitative, and mixed methods studies) and can be used to appraise the quality of empirical studies, i.e., primary research based on experiment, observation, or simulation. The MMAT is widely used; it has been refined since its inception and is a well-accepted tool for the assessment of quality and risk of bias which has been content validated and tested for reliability [32]. It has been used for at least 100 systematic mixed studies reviews worldwide [33]. The appraisal will identify the type of study, the methodology, selection of participants, data collection method, and analysis method. Any disagreements will be resolved through consensus, involving a third reviewer (RW) when needed. Given the lack of consensus on the use of quality appraisal results in qualitative research synthesis [34], no studies will be excluded on the basis of quality assessment. The appraisal will be carried out with the aim of enhancing the transparency of the review.

### 2.8. Data Synthesis

The full texts of included studies will be uploaded on NVivo 12 software. Two sets of syntheses will be conducted, one relating to factors influencing oral health behaviours (research question 1), the second relating to the factors influencing access to, and delivery of, dental care (research question 2 and 3). As these research questions can be addressed by both quantitative and qualitative methods, an integrated approach to synthesis will be taken with quantitative and qualitative data synthesized together through data transformation using a convergent integrated approach [35]. Data from quantitative studies and the quantitative aspects of mixed methods studies will be converted into textual descriptions. Initially, preliminary syntheses of the quantitative data and the qualitative data will be conducted separately. The textual descriptions from the qualitative data will then be integrated with qualitative data for analysis. The narrative synthesis will draw on the framework and techniques described in ‘ERSC Guidance on Conducting Narrative Synthesis’ [36].

The syntheses will be conducted collaboratively by two researchers (JE and MP), in consultation with members of the wider team. Thematic synthesis [37] will be used to carry out an inductive analysis of the qualitative data together with the “qualified” quantitative data. Only findings derived directly from the studies will be coded. Thematic analysis will be used to group codes which share common meaning or experience and the main, recurrent or most important descriptive themes in the literature will be identified, allowing the findings of studies/reports to be summarized and grouped. The constant comparative method will be used to ensure translation of concepts from one study to another [38], looking for the similarities and differences between the findings reported in the papers/reports. New analytic themes will be created by exploring whether the various descriptive themes lead to a new interpretation of the findings not explicitly stated in the primary studies. Three sets of analyses will be conducted relating to oral health behaviours, access to dental care and the provision of dental.

The ENTREQ [39] and PRISMA guidelines [30] will be followed for the reporting of the review. The results will be presented as tables and narrative syntheses.

### 2.9. Dissemination

Dissemination of the findings will be through conferences, presentations, and reports to stakeholders from public health, the dental profession, the autistic community, academia, and other relevant organisations. Easy-read reports will be made available. In addition, the results of the systematic review will be submitted as article(s) to peer-reviewed journals. Autistic adolescents, the parents of autistic children and adolescents, oral health and public health professionals, and other stakeholders will be involved in interpretation and dissemination of the review findings.

## 3. Discussion

Establishing good oral health behaviours and preventative dental care in childhood is essential to good oral heath across the life course [26]. Autistic children and young people are experiencing oral health inequalities [40]. It is important that we explore approaches to better understand the oral health needs of autistic children and young people and to identify ways of supporting their oral health. Establishing the state of current evidence can potentially help support informed decision making by policy makers, commissioners, dental care professionals, and organisations supporting the autistic community. This mixed methods review will systematically explore the available literature on the factors influencing the oral behaviours of autistic children and adolescents, those which influence their access to dental care, and how dental care is provided to them. Taking an inclusive approach, it will throw light on the factors influencing the adoption of good oral health behaviours and the use of dental care services from the perspective of autistic children/adolescents, their parents/care givers, those providing support to children and families, dental professionals, and members of the wider dental care team. The review will provide insights into the current state of research in this area, highlighting where research gaps exist. The results will be interpreted in the broader context. We will also seek to interpret the results in the broader context of oral health inequalities.

The protocol for this review has been developed by a team which brings together a wide range of relevant expertise and experience. This includes an information specialist, experts in dental public health, community engagement and research, a community dentist, a young autistic person, and a researcher who is also a leading figure in the local autism community. Having this team not only strengthens the project, but will also facilitate the sharing of relevant findings through a broad local and national network.

The NHS Long Term Plan aims to improve the health of people with a learning disability, people who are autistic, or both, and this includes oral health [41]. This review is part of a wider study, working together with autistic children/adolescents, parents/carers, support staff, and dental care professionals to explore the factors influencing the oral health behaviours of autistic children/adolescents, their access to dental care services and its provision, and how barriers can be overcome. The findings will be used in the development of an appropriate local clinic care pathway for autistic children/adolescents, and to inform national policies and practices.

## 4. Conclusions

This systematic review will collate evidence on the factors influencing autistic children and adolescents’ adoption of beneficial oral health behaviours and access to dental care. It will also explore those factors influencing provision of dental care to this population. By bringing together and analysing the evidence from qualitative, quantitative and mixed methods studies it will contribute to a greater understanding of the oral health inequalities experienced by autistic people. 

## Figures and Tables

**Table 1:** Summary of Inclusion and Exclusion Criteria.

Study Characteristic	Inclusion Criteria	Exclusion Criteria
Population	Studies involving: autistic children and adolescents 19 years of age or under at the time of the study. Studies addressing both adults and children will be included if the data/findings relating to children are clearly defined and reported separately. Parents/guardians/caregivers, and support staff (e.g., support workers, volunteers, and teachers), who must be caring for, working with, or supporting at least one autistic child or adolescent. Oral health care providers to include all individuals involved in the provision of dental care or promotion of oral health, e.g., dentists, dental hygienists, dental nurses, oral health educators, and other members of the wider dental team, such as receptionists.	Studies involving autistic individuals aged 20 and above at the time of the study.
Setting	Studies from countries with a Human Development Index value of 0.8 or above	Studies from countries with a Human Development Index value below 0.8
Outcomes	Studies that include outcomes relating to: factors influencing oral health behaviours in autistic children and adolescents. Factors influencing access to dental care services by autistic children and adolescents. Factors influencing the provision of dental care to autistic children and adolescents by oral health care providers.	Studies which do not include outcomes relating to the factors influencing oral health behaviours in autistic children and adolescents, their access to dental care or the provision of dental care to them.

## Data Availability

Not applicable.

## Appendix A

Countries with Very High Human Development Index.

Andorra	Korea (Republic of)
Argentina	Kuwait
Australia	Latvia
Austria	Liechtenstein
Bahamas	Lithuania
Bahrain	Luxembourg
Barbados	Malaysia
Belarus	Malta
Belgium	Montenegro
Brunei Darussalam	Netherlands
Bulgaria	New Zealand
Canada	Norway
Chile	Oman
Croatia	Poland
Cyprus	Portugal
Czech Republic	Qatar
Denmark	Romania
Estonia	Russian Federation
Finland	Saudi Arabia
France	Singapore
Germany	Slovakia
Greece	Slovenia
Hong Kong, China (SAR)	Spain
Hungary	Sweden
Iceland	Switzerland
Ireland	United Arab Emirates
Israel	United Kingdom
Italy	United States
Japan	Uruguay
Kazakhstan	

## Appendix B

**Search strategies**

Search strategy for EMBASE

Embase <1974 to 12 February 2021>

1. exp Autism/74474
2. Autis*.ab,kw,ti. 68519
3. Pervasive development disorder*.ab,kw,ti. 141
4. Kanner*.ab,kw,ti. 332
5. (Asperg* not aspergill*).ab,kw,ti. 3761
6. Rett.ab,kw,ti. 4736
7. 1 or 2 or 3 or 4 or 5 or 6 86514
8. dental health/4094
9. dental procedure/26330
10. exp tooth disease/220856
11. exp dentist/25749
12. (oral adj3 (health* or hygiene or care)).ab,kw,ti. 48814
13. dental.ab,kw,ti. 228647
14. ((tooth adj3 (health* or hygiene or care or brush* or floss*)) or toothbrush*).ab,kw,ti. 8182
15. (teeth adj3 (health* or hygiene or care or brush* or floss*)).ab,kw,ti. 4245
16. dentist*.ab,kw,ti. 74090
17. or/8–16 439135
18. 7 and 17 803

Ovid MEDLINE(R) and Epub Ahead of Print, In-Process & Other Non-Indexed Citations, Daily and Versions(R) <1946 to 15 February 2021>

1. exp Autism Spectrum Disorder/ 31735
2. Autis*.ab,kw,ti. 52514
3. Pervasive developmental disorder*.ab,kw,ti. 2048
4. Kanner*.ab,kw,ti. 227
5. (Asperg* not aspergill*).ab,kw,ti. 2384
6. Rett.ab,kw,ti. 3632
7. 1 or 2 or 3 or 4 or 5 or 6 59204
8. Oral Health/ 17243
9. Oral Hygiene/ 13093
10. Dental Care/ 21609
11. dental health services/ 4127
12. exp Tooth Diseases/ 175921
13. Dentists/ 18313
14. (oral adj3 (health* or hygiene or care)).ab,kw,ti. 44013
15. dental.ab,kw,ti. 225529
16. ((tooth adj3 (health* or hygiene or care or brush* or floss*)) or toothbrush*).ab,kw,ti. 7995
17. (teeth adj3 (health* or hygiene or care or brush* or floss*)).ab,kw,ti. 3806
18. dentist*.ab,kw,ti. 77372
19. 8 or 9 or 10 or 11 or 12 or 13 or 14 or 15 or 16 or 17 or 18 414449
20. 7 and 19 494

Database—CINAHL Plus with Full Text

S1 ((MH “Asperger Syndrome”) OR (MH “Autistic Disorder”) OR (MH “Pervasive Developmental Disorder-Not Otherwise Specified”)) OR TI (Autis* or Asperg* or “pervasive developmental disorder” or Rett or Kanner) OR AB (Autis* or Asperg* or “pervasive developmental disorder” or Rett or Kanner) OR SU (Autis* or Asperg* or “pervasive developmental disorder” or Rett or Kanner) 37,553
S2 (MH “Oral Health”) 12,752
S3 (MH “Oral Hygiene”) 5937
S4 (MH “Dental Care+”) 18,517
S5 (MH “Dental Health Services”) 1424
S6 (MH “Tooth Diseases+”) 35,558
S7 (MH “Dentists”) 10,488
S8 TI ((oral N3 (health* or hygiene or care))) OR AB ((oral N3 (health* or hygiene or care))) OR SU ((oral N3 (health* or hygiene or care))) 27,688
S9 TI dental or dentist* OR AB dental or dentist* OR SU dental or dentist* 136,992
S10 TI (((tooth N3 (health* or hygiene or care or brush* or floss*)) or toothbrush*)) OR AB (((tooth N3 (health* or hygiene or care or brush* or floss*)) or toothbrush*)) OR SU (((tooth N3 (health* or hygiene or care or brush* or floss*)) or toothbrush*)) 5669
S11 TI ((teeth N3 (health* or hygiene or care or brush* or floss*))) OR AB ((teeth N3 (health* or hygiene or care or brush* or floss*))) OR SU ((teeth N3 (health* or hygiene or care or brush* or floss*))) 2398
S12 S2 OR S3 OR S4 OR S5 OR S6 OR S7 OR S8 OR S9 OR S10 OR S11 154,615
S13 S1 AND S12 377

Database—Dentistry & Oral Sciences Source

S1 DE “AUTISM” OR DE “ASPERGER’S syndrome” OR DE “AUTISM in adolescence” OR DE “AUTISM in adults” OR DE “AUTISM in children” 161
S2 DE “PERVASIVE child development disorders” 5
S3 TI (Autis* or Asperg* or “pervasive developmental disorder” or Rett or Kanner) OR AB (Autis* or Asperg* or “pervasive developmental disorder” or Rett or Kanner) OR SU (Autis* or Asperg* or “pervasive developmental disorder” or Rett or Kanner) 487
S4 S1 OR S2 OR S3 489
S5 (DE “ORAL health”) OR (DE “ORAL hygiene”) 13,599
S6 (DE “DENTAL care”) OR (DE “DENTISTS”)) OR (DE “DENTAL pathology+”) 35,901
S7 TI ((oral N3 (health* or hygiene or care))) OR AB ((oral N3 (health* or hygiene or care))) OR SU ((oral N3 (health* or hygiene or care))) 33,270
S8 TI (dental or dentist*) OR AB (dental or dentist*) OR SU (dental or dentist*) 244,563
S9 TI (((tooth N3 (health* or hygiene or care or brush* or floss*)) or toothbrush*)) OR AB (((tooth N3 (health* or hygiene or care or brush* or floss*)) or toothbrush*)) OR SU (((tooth N3 (health* or hygiene or care or brush* or floss*)) or toothbrush*)) 12,076
S10 TI ((teeth N3 (health* or hygiene or care or brush* or floss*))) OR AB ((teeth N3 (health* or hygiene or care or brush* or floss*))) OR SU ((teeth N3 (health* or hygiene or care or brush* or floss*))) 9454
S11 S5 OR S6 OR S7 OR S8 OR S9 OR S10 253,069
S12 S4 AND S11 306

Database—SocINDEX

S1 DE “AUTISM” OR DE “AUTISM in adolescence” OR DE “AUTISM in adults” OR DE “AUTISM in children” 1981
S2 TI (Autis* or Asperg* or “pervasive developmental disorder” or Rett or Kanner) OR AB (Autis* or Asperg* or “pervasive developmental disorder” or Rett or Kanner) OR KW (Autis* or Asperg* or “pervasive developmental disorder” or Rett or Kanner) 3827
S3 S1 OR S2 3909
S4 DE “DENTAL care” 624
S5 TI ((oral N3 (health* or hygiene or care))) OR AB ((oral N3 (health* or hygiene or care))) OR KW ((oral N3 (health* or hygiene or care))) 1063
S6 TI (dental or dentist*) OR AB (dental or dentist*) OR KW (dental or dentist*) 4373
S7 TI (((tooth N3 (health* or hygiene or care or brush* or floss*)) or toothbrush*)) OR AB (((tooth N3 (health* or hygiene or care or brush* or floss*)) or toothbrush*)) OR KW (((tooth N3 (health* or hygiene or care or brush* or floss*)) or toothbrush*)) 208
S8 TI ((teeth N3 (health* or hygiene or care or brush* or floss*))) OR AB ((teeth N3 (health* or hygiene or care or brush* or floss*))) OR KW ((teeth N3 (health* or hygiene or care or brush* or floss*))) 148
S9 S4 OR S5 OR S6 OR S7 OR S8 4839
S10 S3 AND S9 4

Database—SCOPUS

(TITLE-ABS-KEY (autis* OR asperg* OR “pervasive developmental disorder” OR rett OR kanner)) AND (TITLE-ABS-KEY (dental OR dentist* OR toothbrush*) OR TITLE-ABS-KEY (oral W/3 (health* OR hygiene OR care)) OR TITLE-ABS-KEY ((tooth OR teeth) W/3 (health* OR hygiene OR care OR brush* OR floss*))) AND NOT TITLE-ABS-KEY (aspergillosis) 855

Database—Web of Science

1. TOPIC: (autis* OR asperger* OR “pervasive developmental disorder” OR rett OR kanner)

2. TOPIC: (dental OR dentist* OR toothbrush*) OR TOPIC: (oral near/3 (health* OR hygiene OR care)) OR TOPIC: ((tooth OR teeth) near/3 (health* OR hygiene OR care OR brush* OR floss*))

1 and 2

433

Database—Psychinfo

(MAINSUBJECT.EXACT.EXPLODE(“Autism Spectrum Disorders”) OR ti(autis* OR asperger* OR “pervasive developmental disorder” OR rett OR kanner) OR ab(autis* OR asperger* OR “pervasive developmental disorder” OR rett OR kanner)) AND ((MAINSUBJECT.EXACT.EXPLODE(“Oral Health”) OR MAINSUBJECT.EXACT.EXPLODE(“Dental Treatment”) OR MAINSUBJECT.EXACT(“Dental Health”) OR MAINSUBJECT.EXACT(“Dentists”)) OR (ti(oral health OR oral hygiene OR oral care) OR ab(oral health OR oral hygiene OR oral care)) OR (ti(tooth health* OR tooth hygiene OR tooth care OR tooth brush* OR tooth floss* OR toothbrush* OR teeth health* OR teeth hygiene OR teeth care OR dental OR dentist*) OR ab(tooth health* OR tooth hygiene OR tooth care OR tooth brush* OR tooth floss* OR toothbrush* OR teeth health* OR teeth hygiene OR teeth care or dental OR dentist*))) 60. Grey literature searches:

**Organisation**	**Web** **Address**	**Search Strings**	**Quick Link to Non-Google Search** **Results**
Public Health England	www.gov.uk (accessed on 21 February 2021)	(“oral health” OR “oral hygiene” OR “oral care” OR dental OR dentist OR toothbrush OR toothbrushing OR teeth) AND (autism OR autistic OR asperger)	https://www.gov.uk/search/all?keywords=(%22 oral+health%22+OR+%22 oral+hygiene%22+OR+%22 oral+care%22+OR+dental+OR+dentist+OR+toothbrush+OR+toothbrushing+OR+teeth)+AND+(autism+OR+autistic+OR+asperger)+&order=relevance (accessed on 21 February 2021)
Health Foundation	www.health.org.uk (accessed on 21 February 2021)	via Google.co.uk: (“oral health” OR “oral hygiene” OR “oral care” OR dental OR dentist OR toothbrush OR toothbrushing OR teeth) AND (autism OR autistic OR asperger OR “pervasive developmental disorder”) AND site: www.health.org.uk (accessed on 21 February 2021)	
National Institute for Health and Care Excellence	nice.org.uk (accessed on 21 February 2021)	(“oral health” OR “oral hygiene” OR “oral care” OR dental OR dentist OR toothbrush OR toothbrushing OR teeth) AND (autism OR autistic OR asperger OR “pervasive developmental disorder”)	https://www.nice.org.uk/search?q=(%22oral%20health%22%20OR%20%22oral%20hy-giene%22%20OR%20%22oral%20care%22%20OR%20dental%20OR%20dentist%20OR%20toothbrush%20OR20toothbrushing%20OR%20teeth)%20AND%20(autism%20OR%20autistic%20OR%20asperger%20OR%20%22pervasive%20developmental%20disorder%22) (accessed on 21 February 2021)
University of Cambridge Autism Research Centre	https://www.autismresearchcentre.com/ (accessed on 21 February 2021)	via Google.co.uk: (“oral health” OR “oral hygiene” OR “oral care” OR dental OR dentist OR toothbrush OR toothbrushing OR teeth) AND site: autismresearchcentre.com (accessed on 21 February 2021)	
National Autistic Society	https://www.autism.org.uk (accessed on 21 February 2021)	via Google.co.uk: (“oral health” OR “oral hygiene” OR “oral care” OR dental OR dentist OR toothbrush OR toothbrushing OR teeth) AND site: autism.org.uk (accessed on 21 February 2021)	
Scottish Autism	https://www.scottishautism.org/ (accessed on 21 February 2021)	via Google.co.uk: (“oral health” OR “oral hygiene” OR “oral care” OR dental OR dentist OR toothbrush OR toothbrushing OR teeth) AND site: scottishautism.org (accessed on 21 February 2021)	
Autistica	https://www.autistica.org.uk/ (accessed on 21 February 2021)	via Google.co.uk: (“oral health” OR “oral hygiene” OR “oral care” OR dental OR dentist OR toothbrush OR toothbrushing OR teeth) AND site: autistica.org.uk (accessed on 21 February 2021)	
British Society for Disability & Oral Health	https://bsdh.org (accessed on 21 February 2021)	via Google.co.uk: (autism OR autistic OR asperger OR “pervasive developmental disorder”) AND site: https://bsdh.org (accessed on 21 February 2021)	
British Society for Pediatric Dentistry	https://www.bspd.co.uk (accessed on 21 February 2021)	via Google.co.uk: (autism OR autistic OR asperger OR “pervasive developmental disorder”) AND site: https://www.bspd.co.uk (accessed on 21 February 2021)	
British Dental Association	https://bda.org (accessed on 21 February 2021)	via Google.co.uk: (autism OR autistic OR asperger OR “pervasive developmental disorder”) AND site: https://bda.org (accessed on 21 February 2021)	
